# Silver Nanoparticles from *Annona muricata* Peel and Leaf Extracts as a Potential Potent, Biocompatible and Low Cost Antitumor Tool

**DOI:** 10.3390/nano11051273

**Published:** 2021-05-12

**Authors:** María G. González-Pedroza, Liliana Argueta-Figueroa, René García-Contreras, Yaiza Jiménez-Martínez, Eduardo Martínez-Martínez, Saúl A. Navarro-Marchal, Juan A. Marchal, Raúl A. Morales-Luckie, Houria Boulaiz

**Affiliations:** 1 Department of Biotechnology, Faculty of Sciences, Autonomous University of the State of Mexico (UAEMex), Toluca 50200, Mexico; mggonzalezp@uaemex.mx; 2 Conacyt Chairs, Faculty of Dentistry, Benito Juárez Autonomous University of Oaxaca, Oaxaca 68120, Mexico; l_argueta_figueroa@hotmail.com; 3 Interdisciplinary Research Laboratory, Nanostructures and Biomaterials, National School of Higher Studies (ENES) León Unit, National Autonomous University of Mexico (UNAM), Leon 37684, Mexico; dentist.garcia@gmail.com; 4 Regenerative Institute of Biopathology and Medicine (IBIMER), University of Granada, 18016 Granada, Spain; yaijmartinez@hotmail.com (Y.J.-M.); navarrosa@ugr.es (S.A.N.-M.); jmarchal@ugr.es (J.A.M.); 5 Laboratory of Cell Communication and Extracellular Vesicles, National Institute of Genomic Medicine (INMEGEN), Mexico City 14610, Mexico; emartinez@inmegen.gob.mx; 6 Department of Applied Physics, Faculty of Sciences, University of Granada, 18071 Granada, Spain; 7 Biosanitary Institute of Granada (ibs. GRANADA), SAS—Universidad de Granada, 18016 Granada, Spain; 8 Research Unit “Modeling Nature” (MNat), University of Granada, 18016 Granada, Spain; 9 Department of Human Anatomy and Embryology, University of Granada, 18016 Granada, Spain; 10 Joint Center for Research in Sustainable Chemistry UAEMex—UNAM (CCIQS), Autonomous University of the State of Mexico (UAEMex), Toluca 50200, Mexico

**Keywords:** green synthesis of silver nanoparticles, *Annona muricata* fruit peel, acetogenins, antiproliferative activity

## Abstract

Cancer is one of the most prevalent diseases in the world and requires new therapies for its treatment. In this context, the biosynthesis of silver nanoparticles (AgNPs) has been developed to treat different types of tumors. The *Annona muricata* plant is known for having anticancer activity. Its main compounds present in the leaves, stems and skin, allowing for its use as reducing agents. In this manuscript, AgNPs with leaf extract (AgNPs-LE) and fruit peel extract (AgNPs-PE) of *A. muricata* were biosynthesized obtaining an average nanoparticle diameter sizes smaller than 50 nm, being 19.63 ± 3.7 nm and 16.56 ± 4.1 nm, and with a surface plasmonic resonance (SPR) at 447 and 448 nm, respectively. The lactone functional group present in the LE and PE extracts was identified by the FTIR technique. The behavior and antiproliferation activity of AgNPs-LE and AgNPs-PE were evaluated in breast, colon and melanoma cancer cell lines. Our results showed that *Annona muricata* fruit peel, which is a waste product, has an antitumor effect more potent than leaf extract. This difference is maintained with AgNPs where the destruction of cancer cells was, for the first time, achieved using concentrations that do not exceed 3 μg/mL with a better therapeutic index in the different tumor strains. In conclusion, we present a low-cost one-step experimental setup to generate AgNPs-PE whose in-vitro biocompatibility and powerful therapeutic effect make it a very attractive tool worth exploiting.

## 1. Introduction

Cancer is considered a worldwide public health problem [1]. The International Agency for Research on Cancer estimated that in the years 2018 and 2020, 18.1 and 19.3 million cancers were diagnosed across the world. However, the COVID-19 pandemic has most probably affected the number of cancer diagnoses in many countries, so the actual number of cancers diagnosed in 2020 will likely have been lower. Global estimates also indicate that the number of new cases will increase over the next two decades to 30.2 million new cases per year by 2040. Currently, colon cancer, breast cancer and melanoma are among the cancers with the highest incidence and mortality rates [2]. Conventional treatments such as chemotherapy, radiotherapy, surgery and hormone therapy have certain limitations [3], and research for new and more efficient therapies is required.

Nowadays, a number of innovative cancer treatments are underway and mostly are in preclinical trials, with challenges remaining in administration, effectiveness and safety. Nanotechnology has led to advanced approaches in the screening, diagnosis, and treatment of cancer [4]. Synthesis of noble metal nanoparticles such as gold, copper and silver for applications in the medical area is of constant interest. In this context, silver nanoparticles (AgNPs) are attractive due to their exclusive optical, electrical, magnetic and thermal properties which can be incorporated into antimicrobial applications, biosensor materials, composite fibers, cryogenic superconducting materials, cosmetic products and electronic components. Moreover, AgNPs have been focused on potential applications in cancer diagnosis and therapy [5]. Generally, the synthesis of AgNPs can be carried out using three different methods, including physical, chemical and biological approaches.

The physical method prepares AgNPs by evaporation-condensation techniques using a tube furnace at atmospheric pressure. It’s a speed synthesis process based on the use of radiation as a reducing agent and no hazardous chemical involvement. However, low performance, high energy consumption, solvent contamination, and lack of uniform distribution limit its use [6]. Chemical methods use water or organic solvents in the synthesis of AgNPs [7,8]. They generally employ three main components, such as metal precursors, reducing agents, and stabilizing/protecting agents. Basically, the reduction of silver salts involves two stages: nucleation and growth [9]. It is characterized by its high performance. However, the manufactured particles lack the expected purity, having sedimentation of chemical products on their surfaces. Moreover, it is very difficult to prepare AgNPs with a well-defined size, which requires one more step to prevent particle aggregation. On the other hand, it’s an extremely expensive method that causes, during the synthesis process, the formation of too many toxic and dangerous by products [6].

Biological methods overcome these limitations. In fact, currently, production of nanoparticles of defined size using different biological systems including bacteria, fungi, plant extracts and small biomolecules (e.g., vitamins and amino acids) is an alternative method for the synthesis of various nanoparticles such as gold, graphene and AgNPs [10,11,12]. Green synthesis of AgNPs, based on exploiting the natural capabilities of plant secondary metabolite extract of leaves, roots, stems or, from the shell of a plant to a complete organism, has advantages over routine physical and chemical synthetic approaches. Its one-step experimental setup to reduce and stabilize bulk silver in AgNPs, biocompatible nature, therapeutic importance and low cost make it a very attractive tool worthy of being exploited [13].

In this context, Soursop (*Annona muricata*), is a plant well known for its anticancer activity [14]. This plant is a species of the genus *Annona*, of the family *Annonaceae*, order *Magnoliales* and division *Magnoliophyta* [15]. This tree tends to bloom and bear fruit most of the year. It is distributed in the tropical regions of Central and South America, West Africa and Southeast Asia [16], the *A. muricata* fruit is an edible collective ovoid berry, dark green in colour [17]. The active compounds of *A. muricata* are acetogenins and polyphenols, among others [18,19], and can be used as reducing agents for the biosynthesis of nanoparticles [20]. It should be noted that the anticancer activity is directly attributed to acetogenins, specifically the lactone functional group. Lactones are an organic compound of the cyclic ester type. This functional group is responsible for blocking the complex I at the mitochondrial level in cancer cells, creating the accumulation of protons through the mitochondrial membrane, stopping production of ATP and, therefore, forcing selective apoptosis [21].

At present, several studies have demonstrated the antitumor properties of *A. muricata*, focusing above all on its leaves as it has been traditionally considered “the cancer killer” in conventional medicine [22,23,24]. Taking into account that acetogenins predominate in leaves (LE) and peel (PE) of fruit of *A. muricata*, we used their extracts to synthesize AgNPs-LE and AgNPs-PE to explore and compare their effectiveness and selectivity against different types of tumors.

## 2. Materials and Methods

### 2.1. Collection and Conservation of Leaves and Peel of A. muricata

The leaves and fruits of *A. muricata* were collected in the state of Morelos, in the municipality of Xochitepec (18°46′52″ N, 99°12′02″ W), at the beginning of March, being the month in which the botanical maturity of the fruit is reached. The leaves and fruits of *A. muricata* were characterized in the Herbarium of the Faculty of Sciences, in the UAEMex, State of Mexico, Mexico. Regarding the fruit, it is separated from the peel. Then the leaves and peel of fruit were washed three times with distilled water to remove impurities, and allowed to air dry at room temperature (20 °C) for 15 days.

### 2.2. Biosynthesis of Silver Nanoparticles with Extracts of A. muricata

#### 2.2.1. Preparation of Aqueous Leaf Extract (LE) and Fruit Peel (PE) from *A. muricata*

The leaves and the peel of the fruits were crushed in a mortar until obtaining a homogeneous powder with a size (grain) of 2–3 mm. The extract solutions were prepared by boiling 1 g of the dry fruit peel in a 100 mL Erlenmeyer flask of distilled water for ten minutes at 100 °C. The extract was filtered using filter paper (Whatman No. 1) [25]. A second filtration was carried out with tubes of 30 k Amicon Ultra-15 Centrifugal filter devices, at 300 rpm for 10 min. Then, crude extract was obtained via the concentration of extract with the use of reduced pressure. The same process was used to prepare the leaf extract. This extract can be stored at 4 °C for later use.

#### 2.2.2. Synthesis of Silver Nanoparticles

An experimental analysis was carried out, where different tests were performed varying the volume proportions of extracts and silver nitrate (1 × 10^−2^ M) Sigma-Aldrich, ACS reagent, ≥99.0%, which were 1:2, 1:1 and 2:1; The best concentration was determined by UV-Vis and it was found that, in both cases, the best concentrations were 1:1, consecutively. Subsequently, tubes of 30 k Amicon Ultra-15 Centrifugal filter devices were filtered, at 300 rpm for 10 min, to achieve purification of the generated nanoparticles. The synthesis was carried out as described below. The extract was used for reduction of Ag^+^ ions to Ag^0^, where 5 mL of shell extract and leaves were separately added to 5 mL of aqueous solution of silver nitrate at a concentration of 1 mM. The same process was performed to prepare the silver nanoparticles using leaf extract. Silver ions were reduced to metallic silver in 6 h.

### 2.3. Characterization

#### 2.3.1. Characterization of Optical Properties of AgNPs

The optical properties of the AgNPs were determined using UV-Vis spectroscopy, with the Varian Cary 5000 double-beam equipment, diluting of the silver nanoparticles in deionized H_2_O until the equipment cell was filled. It was analyzed in a range of 200 to 800 nm, in absorption mode.

#### 2.3.2. Morphological and Structural Characterization of AgNPs

The structural properties of the formed nanostructures were characterized, such as morphology, particle size and distribution thereof; for this a JEOL-2100 200 kV Transmission Electron Microscope (TEM) was used with filament of LaB_6_. Placing a drop of the AgNPs sample on a rack until dry, it was subsequently introduced to the sample holder and assembled in the equipment.

Likewise, a Scanning Electron Microscope (SEM) was used to do a chemical mapping of the main elements present, in addition to performing an EDS analysis to determine and identify them.

#### 2.3.3. Characterization of the Main Functional Groups Present in the Extracts of *Annona muricata* and AgNPs

With the help of the FTIR technique and the Bruker TENSOR 27 model equipment, the main functional groups that are present in the extract were recognized, in addition to justifying the presence of AgNPs. It was achieved by placing a drop on the analysis device and operating in transmittance mode from 600 to 4000 cm^−1^.

#### 2.3.4. Characterization of the Zeta Potential

The hydrodynamic mean diameter of the NC was determined by photon correlation spectroscopy (PCS), using a 4700 C light-scattering device (Malvern Instruments, London, UK) and working with a He-Ne laser (10 mW). The light scattered by the samples was detected at 173°, and the temperature was set at 25 °C. The diffusion coefficient measured by dynamic light scattering can be used to calculate the size of the AgNPs-LE and AgNPs-PE by means of the Stokes–Einstein equation. The homogeneity of the size distribution is expressed as polydispersity index (PDI), which was calculated from the analysis of the intensity autocorrelation function. Electrophoretic mobility (μ_e_) and Z potential was measured after diluting a small volume of the (AgNPs-LE and AgNPs-PE) stock (with a total surface equal to 0.05 m^2^) in 1 mL of the desired buffered solution. It should be noted that all the buffers used in the μ_e_ studies had identical ionic strengths, being equal to 0.002 M. The μ_e_ measurements were made in triplicate using a nano zeta dynamic light-scattering analyzer (Zeta-Sizer Nano Z, Malvern Instruments, UK).

### 2.4. Cell Viability Assay

#### 2.4.1. Cell Lines and Cytotoxicity Assays

Breast cancer (MCF-7, MDA-MB-468), colon cancer (HCT-116), Melanoma (A-375) and macrophage cell lines come from the Biobank (Sistema Sanitario Publico Andaluz, Granada, Spain). Cells were grown in Dulbecco’s Modified Eagle’s Medium (DMEM) (Sigma, St. Louis, MO, USA) supplemented with 10% fetal bovine serum (FBS) and 1% penicillin/streptomycin (P/S) (Sigma, St. Louis, MO, USA). The cells (5 × 10^3^ cells) were incubated in 96-well plates at 37 °C in a 5% CO_2_ atmosphere and were treated with different concentrations of biosynthesized nanoparticles AgNPs-PE and AgNPs-LE (100, 50, 25, 12.5, 6.25, 3125, 1556 µg/mL) and different concentrations of extracts PE and LE (2000, 1750, 1500, 1250, 1000, 750, 500, 250 µg for 3 days. Then, the medium was removed and 100 μL of 3-(4,5-dimethyl-2-thiazolyl)-2, 5-diphenyl-2-tetrazoyl bromide (MTT) (at a concentration of 0.2 mg/mL) was added to each well and incubated for three hours. Subsequently, the MTT reagent was removed, and the formazan crystals formed were dissolved in 100 μL of dimethyl sulfoxide (DMSO) and analyzed at 570 nm in a multi-well ELISA plate reader. The inhibitory concentration 50 (IC_50_) was calculated with the GraphPad Prism program (GraphPad 6 Software San Diego, CA, USA). All of the experiments were plated in triplicate and were carried out at least twice. In addition, non-treated cells were used as controls.

#### 2.4.2. Morphological Findings

Morphological findings were assessed using SEM. The cells were grown on sterile coverslips, induced for 6 days with the IC_50_ concentration of the extracts (PE and LE) and nanoparticles (AgNPs-PE and AgNPs-LE), and treated as described by Cáceres et al., 2019 [26]. A Hitachi S-800 scanning electron microscope (Hitachi, Tokyo, Japan) was used for observations. Counting the number of pores per cell was performed using ImageJ with quantification for each cell type from 8 random photos from three independent sample preparations.

#### 2.4.3. Statistical Analysis

The collection of the data from the different biological studies represents the mean ± standard deviation. Two-tailed Student *t*-test was used to compare differences between two groups. A two-tailed *p*-values < 0.05 was considered statistically significant.

## 3. Results and Discussion

### 3.1. Synthesis of Silver Nanoparticles (AgNPs)

In the context of the generation of nanoparticles and as part of a bottom-up method, different variables for the formation of AgNPs must be recognized such as: temperature, reaction time, concentration of extracts and precursors of metal ions such as AgNO_3_, etc. Therefore, it was determined to perform a green synthesis, which guarantees in many ways the generation of nanoparticles using more bioavailable reducers. Likewise, the synthesis was carried out at room temperature, in addition to using a very low concentration of the precursor salt, which implies low costs, simplicity and a lower environmental impact. On the other hand, Ramachandran et al. have identified some plant metabolites such as ascorbic acid, citric acid, cyclic peptide, ellagic acid, gallic acid, retinoic acid and sorbic acid; these have been identified as compounds responsible for the reduction and stabilization in the synthesis of AgNPs, in addition to providing specific properties with antibacterial, anticancer and antioxidant effects [27].

**Figure 1:**
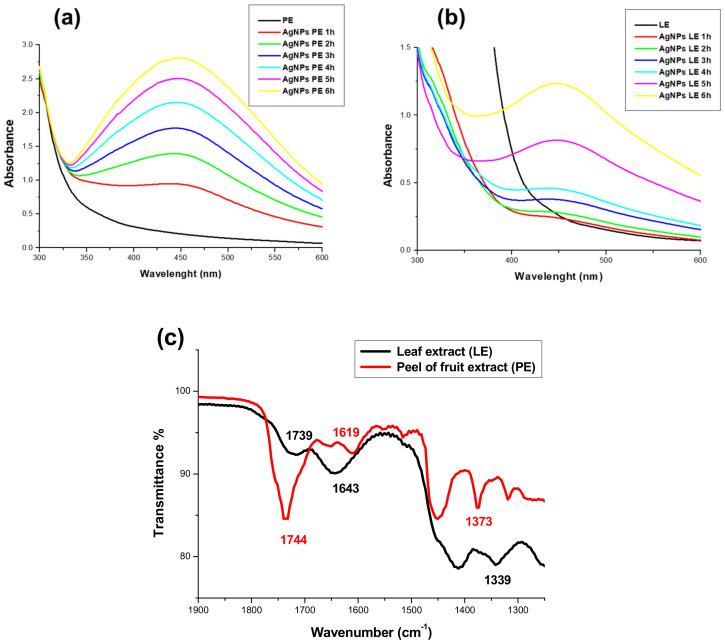
AgNPs-PE and AgNPs-LE characterization: UV-Vis spectra of AgNPs-PE (**a**) and AgNPs-LE (**b**) at different reaction times. FTIR spectrum (**c**) of leaf extracts (LE) and fruit peel extract (PE) of *A. muricata.*

It is important to recognize the characteristic color change of AgNPs, as shown in Figure 1, where leaves and fruit peel extracts undergo a color change as silver ions are reduced, in this case, a yellowish color (the shell extract being a little more amber than the leaf extract) to an intense brown. The majority of authors described a yellowish color on the part of the extracts that, when reacting with AgNO_3_, change specifically from coffee to amber coffee. Santhosh et al. have managed to synthesize AgNPs from leaf extracts of *A. muricata* and describe the reduction of Ag ions by changing the color of the aqueous extract from a yellowish to brown color, in a given time [28].

### 3.2. Spectroscopic Characterization

#### 3.2.1. Spectroscopic Characterization (UV-Vis)

AgNPs offer an optical response known as surface plasmonic resonance (SPR). In addition, the size distribution and shape of AgNPs can be inferred through the amplitude of the band and the position of the maximum absorption band [29]. Based on this rationale, we selected colloidal solutions that had the best volume–volume ratio, both AgNO_3_, as well as extracts. Likewise, the reaction was monitored until observing the maximum absorption band with respect to time, which led to the identification of a six-hour nanoparticle formation period in both cases as shown in Figure 1; after this period of time the behavior is the same because it is the period when the nucleation and growth process of the AgNPs obtained ends. In the case of AgNPs-LE (Figure 1a), the maximum band absorption rate was recorded at 447 nm, while AgNPs-PE (Figure 1b) have a maximum absorption band at 448 nm. Silver nanoparticles usually record a maximum absorption band (RPS) that can appear from 370 nm [30] and up to 500 nm. Some authors, such as Mallirkajuna et al., obtained AgNPs from natural extracts such as *Ocimum sanctum*, registering a maximum absorption band (SPR) at 436 nm [31]; on the other hand, Kumar et al. synthesized AgNPs from *Annona squamosa* shell extracts and reported a band (RPS) at 422 nm [32]; likewise, Vivek et al. obtained AgNPs using *Annona squamosa* leaf extracts reporting a band (RPS) at 444 nm [33].

In addition, it can be seen how the absorbance increases as the reaction time elapses; this is because more AgNPs are generated, and reflects the disappearance of ionic Ag. There are notable differences between AgNPs-PE and AgNPs-LE the absorption band of AgNPs-PE is wider than AgNPs-LE. However, the absorbance is lower in AgNPs-LE, due to the size distribution found in both colloidal solutions. The results agree with what is established in the literature [32,33]: a quasi-spherical morphology and nanoparticles with sizes smaller than 50 nm can be expected in both cases, due to the position of the maximum position band after the 6 h of reaction; likewise, the same maximum absorption band has been observed for three consecutive months. The aqueous *Annona muricata* extracts used have an immense amount of metabolites like polyphenols, acetogenins, citric acid, etc. [13], some of which have already been reported as stabilizers of AgNPs in some other articles [34,35]. Jadhav and collaborators (2016) describe it as a layer that is responsible for stabilization, avoiding agglomeration and precipitation [36].

#### 3.2.2. Spectroscopic Characterization (FTIR) and Z Potential

The FTIR spectrum, shown in Figure 1c, shows a characteristic absorption band at 1744 cm^−1^ (PE) and 1739 cm^−1^ (LE) characteristic of the vibrational mode of conjugated group C=O (stretching), suggesting the presence of a γ-lactone-α, β-unsaturated that distinguishes the vast majority of acetogenins [37,38]. Likewise, the vibrational mode corresponding to the groups C=C (stretching) in 1619 cm^−1^ (PE) and 1643 cm^−1^ (LE) and C-O (stretching) in 1373 cm^−1^ (PE) were recognized and 1339 cm^−1^ (LE), characteristic of the acetogenin molecule.

By the nature of the synthesis of the AgNPs, the AgNPs were expected to be surrounded by the extracts. This was confirmed by the μ_e_ and Z potential with values of−3.2 µmcm/Vs and −34.4 mV, respectively, for AgNPs-PE, while the same parameters for AgNPs-LE was 1.3 µmcm/Vs and 23.3 mV, respectively. This effectively means that the nanoparticles could be surrounded by molecules such as acetogenins, among other metabolites, which help to maintain the stability of nanoparticles in aqueous solution [39].

### 3.3. Morphological and Structural Characterization

#### 3.3.1. Elemental Analysis (SEM-EDS)

**Figure 2:**
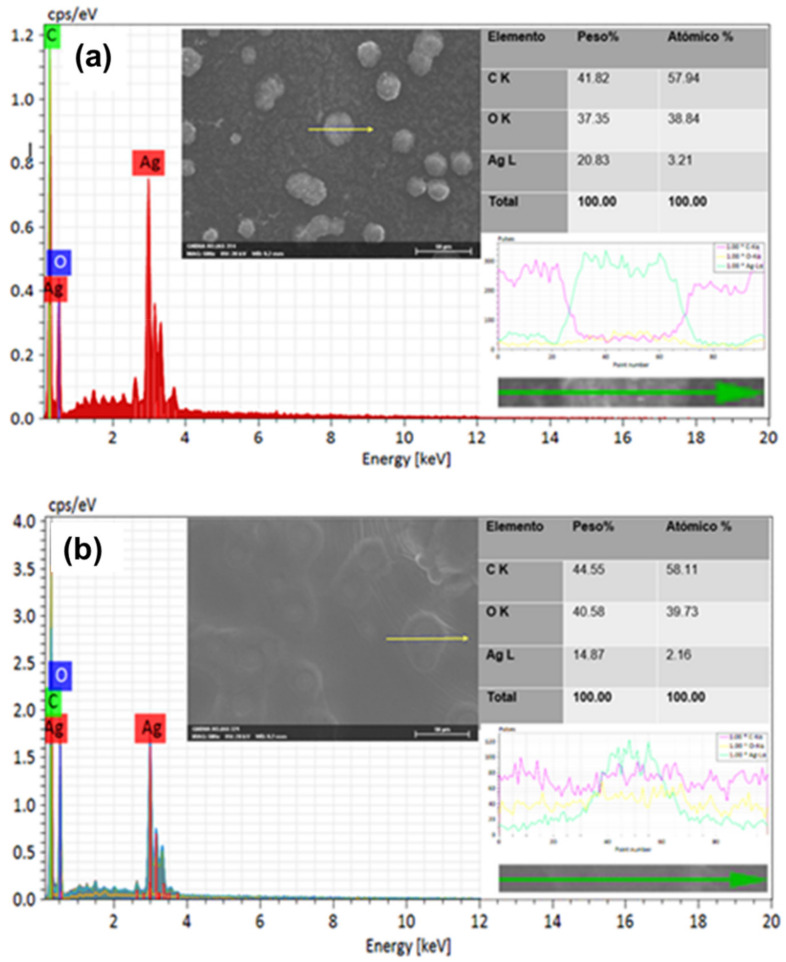
EDS analysis: comparison of the graphs of elementary analysis and cross-line scanning of the generated nanoparticles AgNPs-PE (**a**) and AgNPs-LE (**b**).

**Figure 3:**
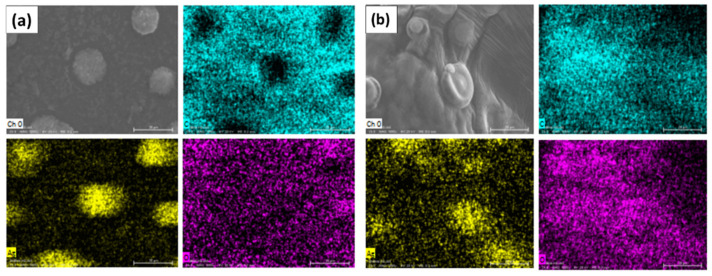
Chemical mapping of the elements carbon (blue color), silver (yellow color) and oxygen (purple color), (**a**) AgNPs-PE and (**b**) AgNPs-LE.

An SEM/EDS analysis was carried out to corroborate the presence of the silver element in the AgNPs (LE and PE) obtained from leaf extracts and peel of *A. muricata*, then a comparison of the analysis graphs shows elementary chemical mapping of micrographs and cross-line scanning (Figure 2; Figure 3).

Regarding the micrographs, different agglomerates (clearer and brighter clusters) of nanoparticles can be observed in both images (Figure 2a,b). In the analysis by energy dispersion spectroscopy (EDS), it was possible to confirm the presence of the silver element in the sample, as can be seen in the spectrum of the same figures, while in the composition table, it is indicated that the percentage of silver is significant in both micrographs, both for AgNPs-PE and for AgNPs-LE; this is due to the concentration of AgNO_3_ from which the AgNPs were generated. In the same way, the EDS spectrum of the transverse line scan is shown in both; however, a greater intensity is reflected in the spectrum of AgNPs-PE (Figure 2). This could be due to the amount of nanoparticles present in the selected agglomerate.

In the chemical mapping, the following elementary maps of both nanoparticles (AgNPs-PE and AgNPs-LE) were obtained. In Figure 2 we can observe that in both cases it is revealed that the surface of the entire sample consists of Ag, C and O, as presumably demonstrated in the EDS spectra. The carbon, oxygen and silver maps support the interpretation of the line scan, shown in Figure 2, agreeing that there is a greater intensity of the silver element in the AgNPs-PE. However, it is more than evident that the greater presence of Ag is present in the agglomerates. Likewise, it is observed that in the areas where there is more presence of Ag, and less amount of C and O (Figure 3). Other authors demonstrate the same behavior in their samples [40,41], this could be due to the preparation of the sample, since the AgNPs agglomerate when the sample is dried.

#### 3.3.2. Morphological Analysis (TEM)

Through Transmission Electron Microscopy (TEM), the size and shape of AgNPs previously synthesized with *A. muricata* leaves and fruit peel were elucidated. As can be seen in Figure 3a,b, it is evident that in both cases AgNPs with sizes smaller than 50 nm were obtained, fulfilling one of the main objectives of this investigation, since it offers better optical properties [42] and biocompatibility [43,44]. Currently, Santhosh et al., [28] have already synthesized AgNPs with leaf extracts of *A. muricata*. However, they obtained an average nanoparticle size of up to 103 nm, which is not favorable for the objective application of this research; therefore, it was decided to experiment with different concentrations of both the precursor salt and the extracts. Likewise, Kumar et al. synthesized AgNPs from fruit peel extracts of *A. squamosa*, thus reporting a large polydispersity ranging from 20 to 60 nm [32], which is not viable for the application required in this project [43,44].

**Figure 4:**
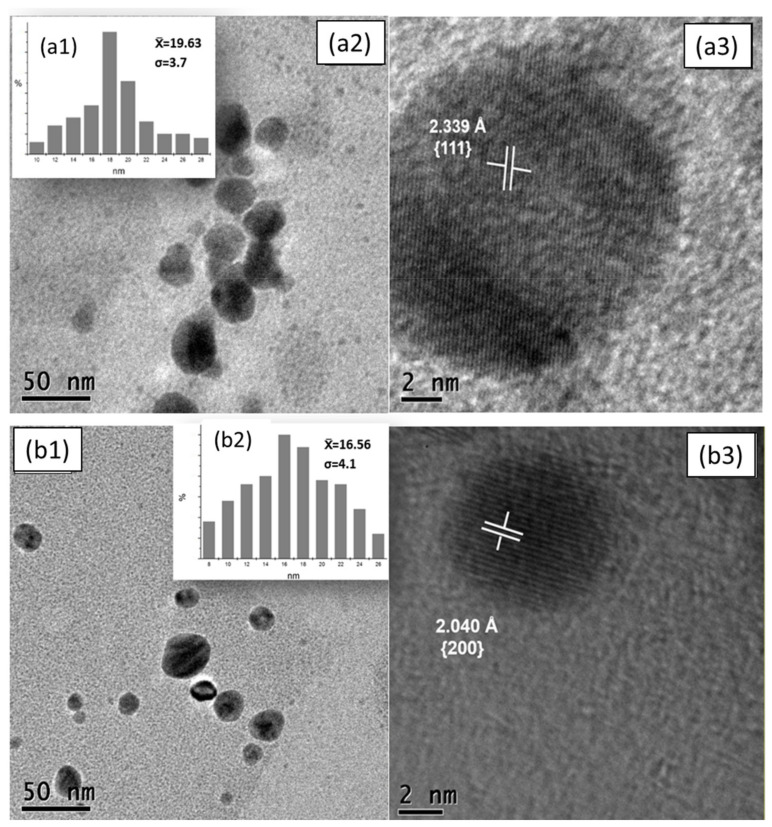
Micrograph obtained by TEM of AgNPs, as well as size distribution graph and HRTEM. The micrographs show the nanoparticles generated from the extracts of leaves and fruit peels of *Annona muricata*. The LE-AgNPs generated show an average diameter size of 19.63 ± 3.7 nm (**a1**) and a quasi-spherical shape (**a2**). The inter-planar spaces with a distance of 2339 Å were determined for the AgNPs-PE which corresponds to the family of planes (111) (**a3**). The LE-AgNPs generated show a quasi-spherical shape, individualized AgNPs can be seen without agglomerations (**b1**). The average diameter size of 16.56 ± 4.1 nm for the AgNPs-PE (**b2**). The inter-planar distances of the AgNPs-LE were measured, obtaining a distance of 2040 Å, which corresponds to the family of planes (200) (**b3**).

It should be noted that quasi-spherical AgNPs-PE and AgNPs-LE were obtained as shown in the micrographs of Figure 4(a2,b1). Other authors report it as irregular spherical or simply spherical forms. The ions capture an electron from the bioreducer and a nucleus or seed is formed. These are formed in the entire solution and there is a “competition” for the growth of the nuclei until the formation of the nanoparticles. Due to this competition between them, which is governed by thermodynamic parameters, a defined size is achieved and when the molecules are at this point they absorb superficially, acting as a protective layer to prevent the nanoparticles from continuing to grow and avoid collisions [27,45]. Likewise, little agglomeration is observed, which is important for the treatment in breast cancer cell lines, since they can be effectively dispersed in the medium.

On the other hand, the measurement of the diameter of the nanoparticles observed in the micrographs was carried out, both of the AgNPs-PE and of the AgNPs-LE by means of the ImageJ software, thus obtaining an average diameter size of 19.63 ± 3.7 nm for AgNPs-LE (Figure 4(a1)) and, with a polydispersity of sizes ranging from 10 to 28 nm. Likewise, the average diameter size of the AgNPs-PE (Figure 4(b2)) was obtained, which turned out to be 16.56 ± 4.1 nm, observing a polydispersity of greater range that goes from 8 to 26 nm. It is evident that the AgNPs-PE have more uniform sizes, even less polydispersity is shown both in histograms and in the same micrographs with respect to the AgNPs in addition, very little agglomeration of AgNPs is recognized in both cases, at the same time observing sizes smaller than 50 nm, thus corroborating the viability for the desired application.

The data obtained from the DLS measurements of hydrodynamic size and polydispersity index (PDI) were 41.16 nm and 0.481 for AgNPs-PE, while the measurements for AgNPs-LE were 48.39 nm and 0.643.

Additionally, an analysis was conducted with HRTEM, with both AgNPs-PE and AgNPs-LE, in order to confirm that both AgNPs were formed by Ag^0^. The inter-planar spaces with a distance of 2339 Å were determined for the AgNPs-PE (Figure 4(a3)), which corresponds to the family of planes (111); in the same way, the inter-planar distances of the AgNPs-LE were measured, obtaining a distance of 2040 Å, which corresponds to the family of planes (200) as shown in Figure 4(b3), taking as reference the JCPDS card 00-004-0783.

### 3.4. Cell Viability Test

**Figure 5:**
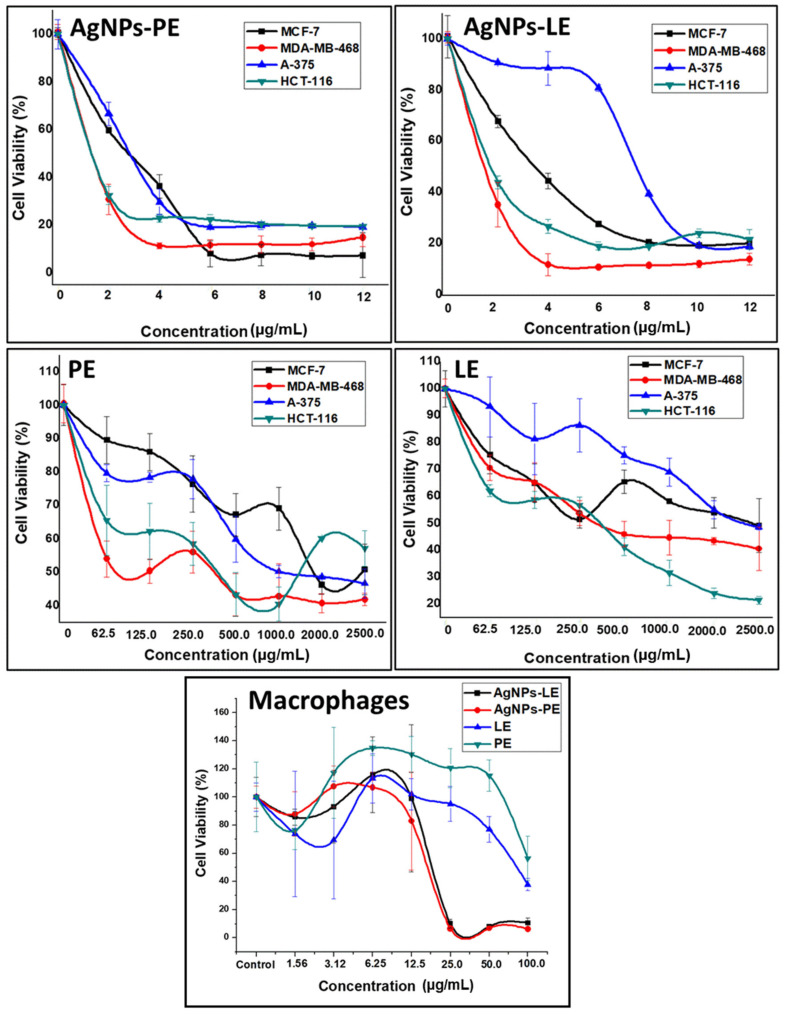
Graphs of the percentage of cell viability with respect to different concentrations of LE, PE, AgNPs-LE and AgNPs-PE.

The percentage of cell viability of the cell lines was determined, compared to different concentrations of AgNPs and extracts by the assay of 3-(4,5-dimethyl-2-thiazolyl)-2, 5-diphenyl-2-tetrazoyl bromide (MTT) (Figure 5). We determined their inhibitory concentration (IC_50_) after 3 days of treatment (Table 1).

The antitumor effect of LE has been previously reported [46]. Fattahi et al. reports an activity of the aqueous extracts against the MCF-7 line with an IC_50_ of 2000 µg/mL relatively similar to those that we have obtained [47]. As shown in Table 1, the IC_50_ values for the PE were lower than LE for all tumor cell lines tested. Indeed, breast cancer cells have been demonstrated to be more sensitive to PE than LE, reducing its IC_50_ by 65.88% and 43.95% for MDA-MB-468 and MCF-7, respectively. In addition, both A-375 melanoma and HCT-116 colon cancer cell lines showed similar sensitivity to PE with 24.14% and 23.6% of IC_50_ decrease in comparison to LE.

On the other hand, our results demonstrated that all cancer cell lines were drastically more sensitive to our biosynthesized AgNPs than extract, as they achieved a better effect with 99% lower concentrations. In this context, several studies demonstrated that the AgNPs had an important anticancer activity. AgNPs synthesized using other plants demonstrated the anti-proliferative activity against several cancer cell lines including human colorectal adenocarcinoma, kidney and cervix [48]. The biosynthesized AgNPs based on *Commelina nudiflora* aqueous extract showed reduced cell viability and increased cytotoxicity in HCT-116 colon cancer cells with IC_50_ concentration of 200 and 100 μg/mL, respectively [49]. Saratale and collaborators prepared AgNPs using *Taraxacum officinale* showing their high cytotoxic effect against human liver cancer cells (HepG2) using an IC_50_ about 60 μg/mL [50]. The *Nostoc linckia* based AgNPs had anticancer activity against breast cancer cell MCF-7 with an IC_50_ of 27.79 μg/mL [51].

To our surprise, our results showed that AgNPs-PE were more potent than AgNPs-LE reaching 64.98%, 37%, 11.78% and 3.63% more effectiveness in HCT-116, A-375, MDA-MB-468 and MCF-7, respectively. In addition, the IC_50_ obtained have been much lower than those described in the literature since with an IC_50_ of only 1.685 µg/mL, our AgNPs-PE from *A. muricata* were able to induce cytotoxicity to MDA-MB-468, a triple negative breast cancer cell line (do not express estrogen receptor (ER), progesterone receptor (PR), and do not have HER-2/Neu amplification), characterized by a poor outcome compared to the other subtypes of breast cancer [52]. To the best of our knowledge, the only study that analyzes the antitumor effect of AgNPs-PE from *A. muricata* was in an AMJ-13 breast cancer cell line (ER^−^,PR^−^ and HER2/neu^+^) and reported an IC_50_ of 17.34 µg/mL using AgNPs synthetized from *A. muricata* stock solution 10-fold more concentrated than ours [53]. This could be due to the geographical origin of the plant, the season in which both the leaves and the peel of the fruit were collected and even its age [54,55,56]. There are specific investigations of the *Annona muricata* species, where significant variations in the biochemical composition are reflected depending on the collection season [57].

A comparison between the tumor cell lines (MCF-7, MDA-MB-468, HCT-116 and A-375) and the macrophage cell line was established in order to define the selective activity of the AgNPs and extract through the determination of the in vitro therapeutic index (TI) (Table 2). The ratio of the toxic dose to the therapeutic dose (In vitro TI = IC_50_ non-tumor cell line/IC_50_ tumor cell line) is defined as the in-vitro TI of a drug [58]. In macrophages we found that the cytotoxic effect of LE extracts was remarkably higher. In fact, our results showed that LE treatment is more cytotoxic for macrophages than for cancer cells. This could be due to alterations in the cytoskeletal function as a result of the treatment with LE and possibly due to the specific function of macrophages. Here the importance of treatment with PE stands out, since at high concentrations there are no indications that it is cytotoxic against macrophages [59]. This could be due to the phytochemicals difference existing between the LE and PE extracts such as phenolic compounds [39], which have reported apoptosis in macrophages [40].

Furthermore, TI was better by far for almost all cell lines treated by AgNPs-PE (Table 2). The best TI was achieved by HCT-116 and MDA-MB-468 (TIs = 10.66 and 8.13 respectively). It should be noted that macrophages are cells of the immune system that are found in tissues and serve as protection against external agents. Our results suggest that macrophages are less susceptible to damage from acetogenins, and it could be that their effect is directly on cancer cells. This is due to the rapid proliferation of cancer cells, since they require high levels of energy, so they may be more sensitive to their decline and have important physiological changes [60]. Some authors attribute the effect of aqueous extracts to the release of NO and TNF-α, which is capable of upregulating the expression of iNOS and TNF-α through the transactivation of NF-κB, and this may be a mechanism by the which this herbal medicine causes its therapeutic effects [61]. The mechanism of action of the acetogenins in cancer cells has been reported in terms of apoptotic cell death and control of the cell cycle checkpoint [62]. Other authors suggest that the presence of AgNPs induces the formation of reactive oxygen species, which are considered the main source of DNA damage [63,64]; it could be then inferred that both acetogenins and AgNPs target cells with the greatest energy need, such as cancer cells, which justifies our results.

**Figure 6:**
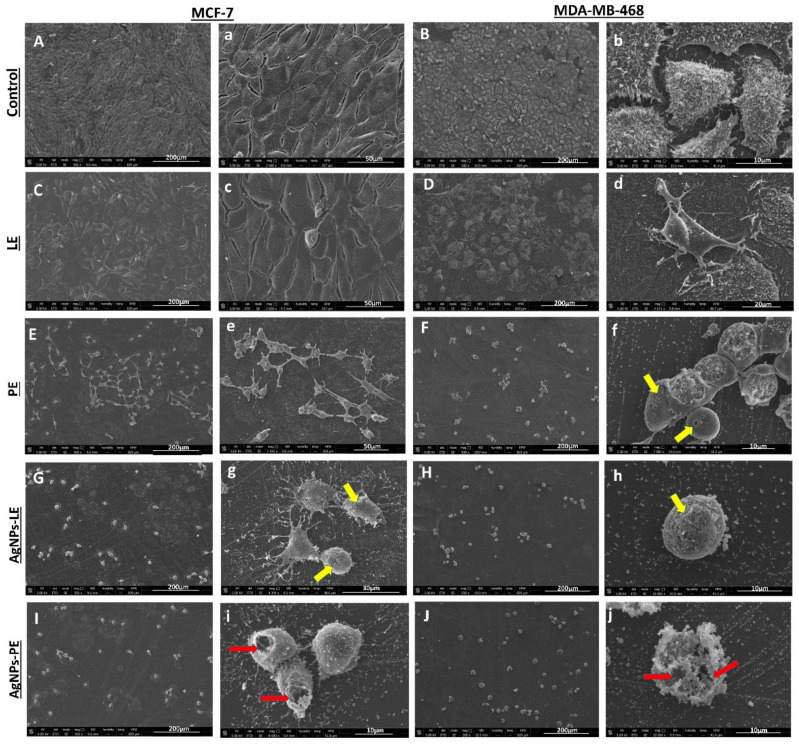
Microscopy micrographs of MCF-7 and MDA-MB-468 cells after 6 days of IC_50_ treatments with different compounds. Cell controls are shown in (**A**,**a**,**B**,**b**) under treatment with LE extract (**C**,**c**,**D**,**d**) showed cells with epithelial form strongly adhered to the surface of the culture coverslip, and PE show cells with loss of microvilli (**E**,**e**,**F**), in addition to multiple pores (arrows yellow) (**f**), the micrographs of the cells treated with AgNPs-LE show a decrease in cells compared to the controls (**G**,**H**). Cells treated with NPs also showed pore formation (yellow arrows, **g**,**h**) and a flatter cell surface than controls, due to the loss of microvilli and filopod structures. Cells treated with AgNPs-PE (**I**,**J**) also show cell decrease, in addition to giant cells with destructured cytoplasmic membrane (red arrows, **i**,**j**) Scale bar (200–10 µm).

After the determination of the anti-tumor activity of the different treatments against the tumor cell lines, we selected MDA-MB-468 and MCF-7 lines, in order to analyze its proliferation and morphological changes after 6 days of induction of different compounds and determine if the use of IC_50_ will achieve total cell death. Our results demonstrated that unlike cells treated with high doses of extracts, lower doses of the AgNPs caused total cell death (Figure 6).

In fact, using SEM we observed that both MCF-7 and MDA-MB-468 control cells were abundant, highly adherent to the substrate and its surface covered by multiple microvillous extensions. However, treatment of MCF-7 and MDA-MB-468 cells with 2280 μg/mL and 776.4 μg/mL of the LE extract, respectively, results in a slight effect on the proliferation of the cell lines- a little more marked in the MDA-MB-468 cells. Even better, the treatment with 1278 μg/mL and 264.9 μg/mL of PE extract has achieved clear cell death with a concomitant decrease in cell density in both MCF-7 and MDA-MB-468 cell lines, respectively. This death has been further accentuated with the use of 2.996 μg/mL and 3.109 μg/mL of AgNPs-LE treatment in both cell lines. Moreover, a similar effect has been observed with the use of even smaller amounts of AgNPs-PE (1.685 μg/mL and 1.910 μg/mL). The treatment with PE, AgNPs-LE and AgNPs-PE showed that only a few round cells remained anchored to the substrate, showing in most of the cases disrupted cytoplasmic membranes and multiple “pores” with different sizes (yellow arrows, Figure 6f). Moreover, cells presented a “flat surface” due to the loss of microvilli and filopodia structures and the apparition of apoptotic bodies a clear signal of death by apoptosis (Figure 6). These results are in concordance with those reported by Jabir and collaborators in THP-1 and AMJ-13 cell lines [53].

Moutin et al. [65] attribute the morphological deteriorations in cells exposed to AgNPs to actin interference with the structure and functions of the cytoskeleton. Damage to the cytoskeleton could result from calcium fluctuations and genetic dysregulation; this creates coherence, since the +ions that are released from the nanoparticles are involved in cell signaling cascades with the activation of Ca^2+^ release that further activates catabolic enzymes and damages mitochondrial membranes. Furthermore, the repeated entry and exit of calcium into the mitochondria could result in damage to the mitochondrial membrane, resulting in the production of ROS and inhibition of ATP synthesis [66]. Knowing the effects that the AgNPs and the acetogenins contained in the extracts have on the breast cancer cell lines, it is easy to adjudge that the cytotoxic effect will be potentiated when the nanoparticles are formed with the extracts, since they will have a duplex function. Likewise, it must be recognized that the cytotoxic effect of the AgNPs will be greater than the extracts due to the concentrations used for their generation.

The reactive oxygen species (ROS) at the cellular level are responsible for inducing DNA damage and, therefore, leading to cell death. Some authors report studies where biosynthesized AgNPs from natural extracts, through redox reactions, where the reducing agent is usually a set of polyphenols present in the extracts, whose function is to cause oxidative stress, which leads to cell death through the mitochondria [67]. Likewise, it is known that the possible mechanism involved in cellular toxicity induced by AgNPs, triggers reactive oxygen species (ROS) by inhibiting the synthesis of intracellular antioxidants [63].

## 4. Conclusions

In this manuscript we demonstrate, for the first time, that even though the leaf of the *Annona muricata* has been traditionally considered “the cancer killer” in conventional medicine, the peel extract of this fruit has an even more powerful antitumor effect. In fact, we were able to produce nanoparticles, with a potent in-vitro antitumor effect, from concentrations of leaves (LE) and peel (PE) of fruit of *A. muricata* extracts much lower than those reported by other authors. Moreover, this difference is accentuated with AgNPs where the destruction of cancer cells was achieved using concentrations that do not exceed 3 μg/mL with a better therapeutic index in the different tumor strains. Our results are very promising and provide the basis for the development of in-vivo experiments for their validation. In conclusion, we present a low-cost one-step experimental setup to generate AgNPs-PE, whose biocompatibility and potent therapeutic effect make it a very attractive tool worthy of being exploited.

## Figures and Tables

**Table 1:** Antiproliferative activities (IC_50_ (µg/mL)) of AgNPs-PE, AgNPs-LE, PE in cancer cell lines and macrophages. All the experiments were performed in triplicate.

Treatment	MCF-7		MDA-MB-468		A-375		HCT-116		Macrophages	
	IC_50_	R^2^	IC_50_	R^2^	IC_50_	R^2^	IC_50_	R^2^	IC_50_	R^2^
AgNPs-PE	2.996	0.9309	1.685	0.9662	2.943	0.9728	1.285	0.9623	13.7	0.9980
AgNPs-LE	3.109	0.9806	1.910	0.9825	8.404	0.9672	2.004	0.9986	10.7	0.9792
PE	1278	0.9592	264.9	0.9676	1880	0.9934	309.3	0.9731	-	-
LE	2280	0.9803	776.4	0.9879	2478	0.9726	404.8	0.9708	68.97	0.9674

**Table 2:** Therapeutic indexes (µg/mL) for AgNPs-PE, AgNPs-LE, PE and LE in different tumor cell lines compared to the macrophage cell line. * ND (not determined).

Treatment	MCF-7	MDA-MB-468	A375	HCT-116
AgNPS-PE	4.34	8.13	4.655	10.66
AgNPS-LE	3.44	5.602	1.273	5.245
PE	ND *	ND *	ND *	ND *
LE	0.030	0.088	0.027	0.170

## Data Availability

Not applicable.
